# Supplementary material for: Complete Genome Sequence of Ovine Mycobacterium avium subsp. paratuberculosis Strain JIII-386 (MAP-S/type III) and Its Comparison to MAP-S/type I, MAP-C, and M. avium Complex Genomes
Source: Microorganisms. 2020 Dec 29;9(1):70. doi: 10.3390/microorganisms9010070 (PMC7823733; doi:10.3390/microorganisms9010070)
Supplement: Supplementary file 1 [file microorganisms-09-00070-s001.zip › Figure_S1_Comparative_alignments_of_five_MAP-C_genome_sequences.pdf]

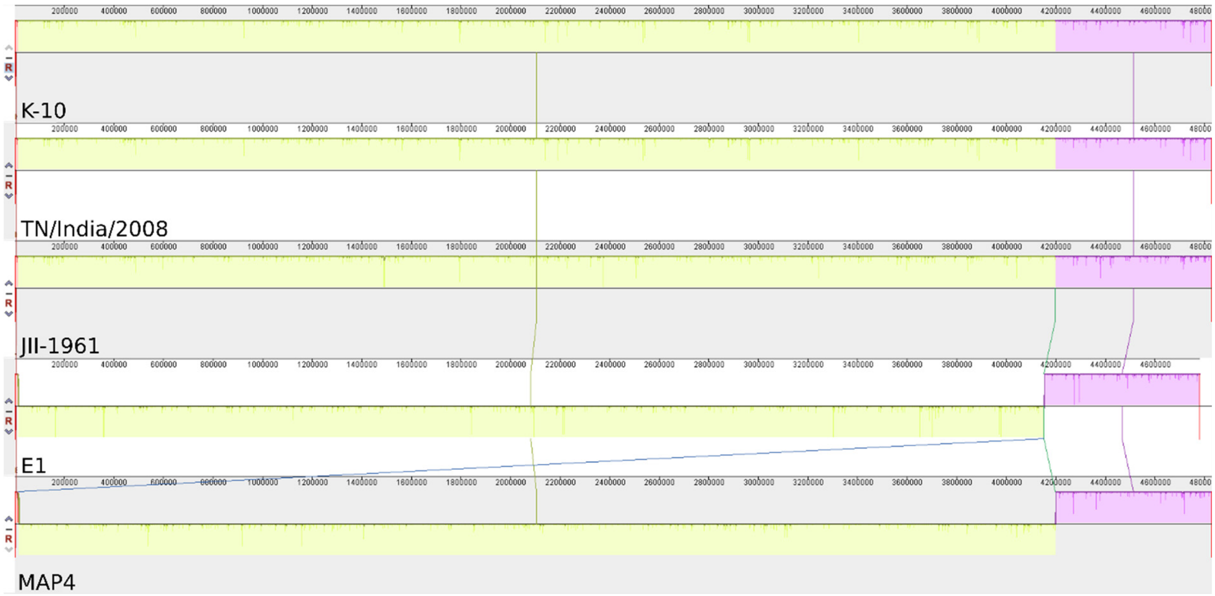

Figure S1. Comparative alignments of five MAP-C-genome sequences using MAUVE 2.4 [53]. The scale is in base pairs.
