# Supplementary material for: Complete Genome Sequence of Ovine Mycobacterium avium subsp. paratuberculosis Strain JIII-386 (MAP-S/type III) and Its Comparison to MAP-S/type I, MAP-C, and M. avium Complex Genomes
Source: Microorganisms. 2020 Dec 29;9(1):70. doi: 10.3390/microorganisms9010070 (PMC7823733; doi:10.3390/microorganisms9010070)
Supplement: Supplementary file 1 [file microorganisms-09-00070-s001.zip › Table_S6_Number_of_different_IS_elements.docx]

**Table S6.** Number of copies of different IS elements in JIII-386, Telford and K-10***.***
Transposable elements belonging to six families were identified and primarily designated by ISfinder software and in addition by genome annotation (PGAP, NCBI).

| **Designation ^1^** | **JIII-386** | **Telford** | **K-10** |
| --- | --- | --- | --- |
|  |  |  |  |
| IS900_IS900_unknown_IS110_ORF_Transposase | 18 | 22 | 17 |
| ISMpa1_ISMpa1_unknown_IS110_ORF_Transposase | 3 | 3 | 3 |
| IS1547_IS1547_unknown_IS110_ORF_Transposase | 2 | 2 | 2 |
| IS1110_IS1110_unknown_IS110_ORF_Transposase | 1 | 1 | 1 |
| IS110 family transposase CDS ^2^ | 1 | 1 | 1 |
|  |  |  |  |
| IS1311_IS1311_unknown_IS256_ORF_Transposase | 9 | 9 | 7 |
|  |  |  |  |
| ISMav2_ISMav2_unknown_IS481_ORF_Transposase | 3 | 3 | 2 |
| ISMav4_ISMav4_unknown_IS481_ORF_Transposase | 2 | 2 | 4 |
| ISMav5_ISMav5_unknown_IS481_ORF_Transposase | 4 | 4 | 3 |
|  |  |  |  |
| ISMgi2_ISMgi2_unknown_IS1182_ORF_Transposase | 7 | 7 | 6 |
|  |  |  |  |
| ISMysp3_ISMysp3_IS51_IS3_ORF_2_Transposase | 3 | 4 | 1 |
|  |  |  |  |
| ISBlo4_ISBlo4_unknown_IS30_ORF_Transposase | 2 | 2 | 2 |
|  |  |  |  |
| total | 55 | 60 | 49 |

^1^ The entries describe the ontology of the database, ^2^ Designation of additionally identified unknown IS110 family transposase CDSs from NCBI annotation.
